# Refinement of an ovine-based immunoglobulin therapy against SARS-CoV-2, with comparison of whole IgG versus F(ab′)_2_ fragments

**DOI:** 10.1038/s41598-023-40277-4

**Published:** 2023-08-25

**Authors:** Stephen Findlay-Wilson, Linda Easterbrook, Sandra Smith, Neville Pope, Matthew Aldridge, Gareth Humphries, Holger Schuhmann, Didier Ngabo, Emma Rayner, Ashley Otter, Thomas Coleman, Bethany Hicks, Rachel Halkerston, Kostis Apostolakis, Stephen Taylor, Susan Fotheringham, Amanda Horton, Irene CanoCejas, Matthew Wand, Julia A. Tree, Mark Sutton, Victoria Graham, Roger Hewson, Stuart Dowall

**Affiliations:** 1 UK Health Security Agency (UKHSA), Porton Down, Salisbury, Wiltshire, SP4 0JG UK; 2 International Therapeutic Proteins Ltd, Longford, TAS 7301 Australia; 3 International Therapeutic Proteins Ltd, Goleigh Farm, Selborne, GU34 3SE Hampshire UK; 4 MicroPharm Ltd, Station Road, Newcastle Emlyn, SA38 9BY UK; 5 Native Antigen Company, Langford Locks, Kidlington, Oxford, OX5 1LH UK

**Keywords:** Applied immunology, Immunotherapy, Infection, Infectious diseases, Medical research, Diseases, Infectious diseases

## Abstract

The development of new therapies against SARS-CoV-2 is required to extend the toolkit of intervention strategies to combat the global pandemic. In this study, hyperimmune plasma from sheep immunised with whole spike SARS-CoV-2 recombinant protein has been used to generate candidate products. In addition to purified IgG, we have refined candidate therapies by removing non-specific IgG via affinity binding along with fragmentation to eliminate the Fc region to create F(ab′)_2_ fragments. These preparations were evaluated for in vitro activity and demonstrated to be strongly neutralising against a range of SARS-CoV-2 strains, including Omicron B2.2. In addition, their protection against disease manifestations and viral loads were assessed using a hamster SARS-CoV-2 infection model. Results demonstrated protective effects of both IgG and F(ab′)_2_, with the latter requiring sequential dosing to maintain in vivo activity due to rapid clearance from the circulation.

## Introduction

The outbreak of severe acute respiratory syndrome coronavirus-2 (SARS-CoV-2), resulting in an ongoing global pandemic first declared in March 2020^1^, has created an urgency to develop and assess new interventions. As part of this activity, we have developed purified ovine immunoglobulins against the whole SARS-CoV-2 spike protein, alongside immunoglobulins specific for the individual S1 and S2 subunits, which provide protection against disease onset^2^.

Whilst highly effective in their therapeutic effects, one of the main issues with the antisera and whole IgG therapies is the risks of serum sickness^3,4^ and allergic reaction^5^. In addition, antibodies specific to SARS-CoV have also been demonstrated to show an enhancement effect, particularly noted when given at low levels^6^. This antibody-dependent enhancement has also been further confirmed in SARS-CoV-2^7,8^. Alongside these potentially adverse sequelae, in SARS-CoV-2 infection acute lung injury has been associated with the Fc region of specific anti-spike IgG^9^. To overcome these effects, removal of the Fc region has been undertaken for next-generation passive immunotherapy uses^10–12^.

Fc removal can either produce monovalent (Fab) or divalent (F(ab′)_2_) fragments. The smaller Fab fragments are rapidly eliminated via renal functions^13^ and in clinical studies have been shown to be eliminated 5–7 times faster than F(ab′)_2_^14^. In addition to the benefits of lessening side effects, the smaller size and reduced cell affinity of F(ab′)_2_ fragments enables them to penetrate deeper into tissues, thus enabling activity within extravascular regions^15,16^ and distribution across body compartments, including the lungs^17^. Altogether, these additional properties may confer advantages over the use of convalescent plasma first authorised for emergency use by the U.S. Food and Drug Administration (FDA) at the early stages of the pandemic.

**Figure 1:**
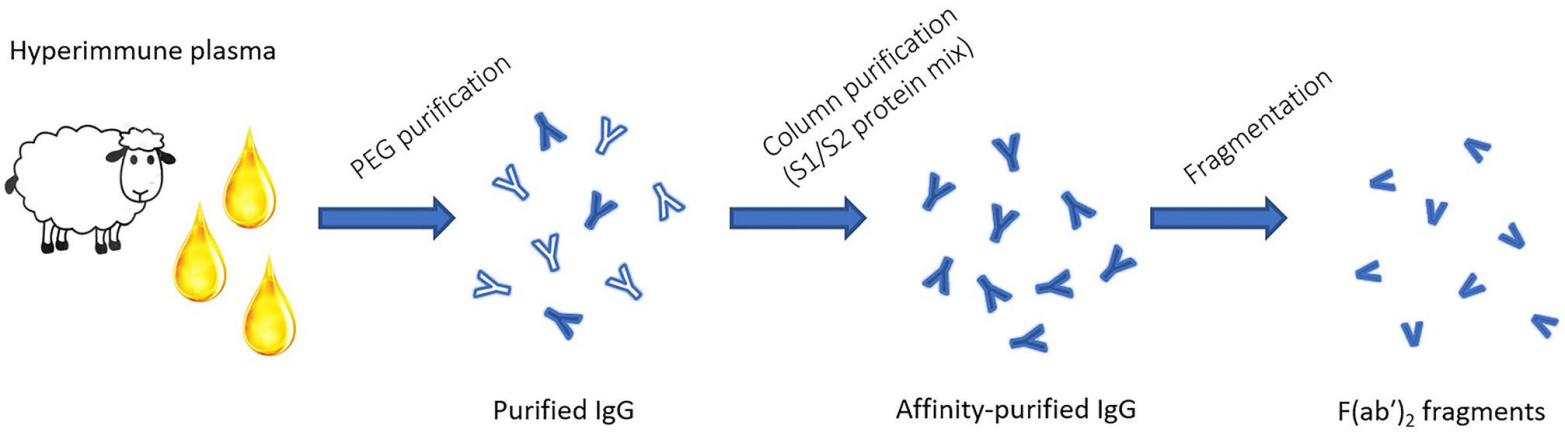
Schematic diagram outline the process for producing purified IgG, affinity-purified IgG and F(ab’2) fragment preparations developed as SARS-CoV-2 therapeutic candidates.

Animal-sourced immunoglobulins have been used widely as antivenoms^18^ and extended to antitoxins^19^. The use of hyperimmune sera to generate antibody-based therapies against infectious diseases has been used for SARS-CoV^20^, MERS-CoV^21^, Ebola^22–24^ and avian influenza virus^25^. During the COVID-19 pandemic, this methodology has been employed by multiple groups, though mainly using horses as the original antibody source^17,26^. In this work, we describe use of an ovine-based approach. Sheep offer a suitable alternative, as horses are regarded as companion animals and are not permitted for antibody production purposes in countries such as the UK^18^. We processed hyperimmune plasma from sheep immunised with whole spike SARS-CoV-2 recombinant protein into three preparations: purified IgG, affinity-purified IgG and F(ab′)_2_ fragments (Fig. 1) which we have then evaluated for in vitro activity before assessing in a developed hamster infection model of SARS-CoV-2 infection^27^.

## Results

### Binding recognition of antibodies and F(ab′)_2_ fragments to recombinant spike SARS-CoV-2 proteins

**Figure 2:**
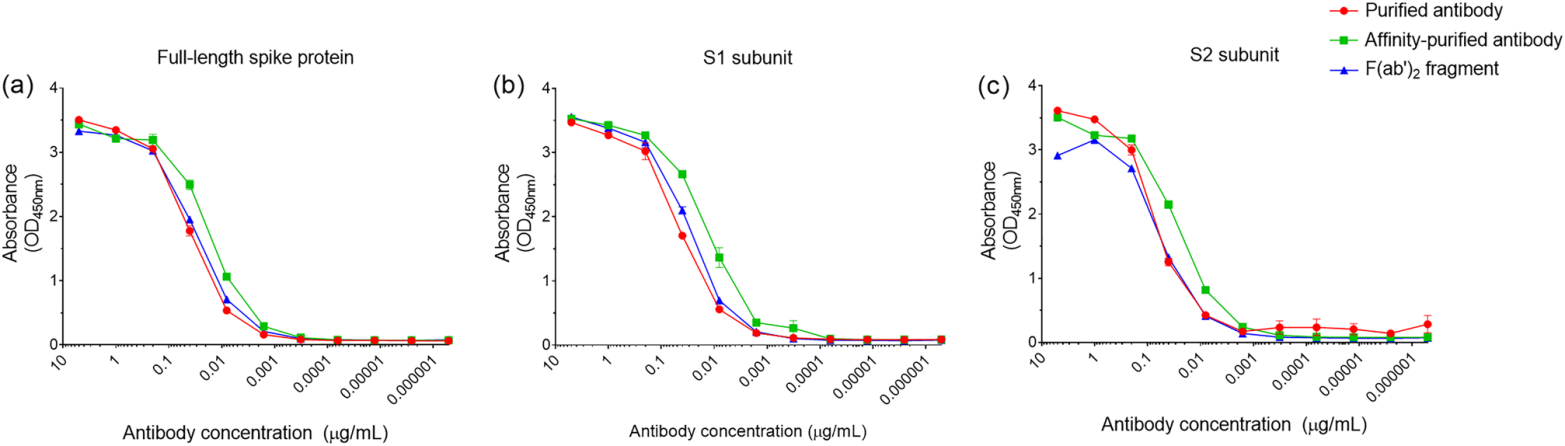
Antigen binding kinetics of purified, affinity-purified and F(ab′)_2_ fragments to recombinant SARS-CoV-2 glycoproteins. (**a**) Reactivity to whole spike protein. (**b**) Reactivity to S1 subunit protein. (**c**) Reactivity to S2 subunit protein. Lines indicate mean values with error bars denoting standard error.

To assess the binding of the preparations after different stages of refinement, ELISA studies were conducted using recombinant spike proteins. Results demonstrated recognition of the antibodies and F(ab′)_2_ fragments to the whole spike protein and recognition across both the S1 and S2 subunits (Fig. 2). Previous ELISAs on ovine sera pre-immunisation demonstrated no cross-reactivity with the recombinant spike protein^2^. As expected, there was higher binding of the affinity-purified preparation compared to the purified preparation due to the removal of non-specific IgG. For the F(ab′)_2_ fragment preparation, binding levels were equivalent to the purified IgG.

### Functional activity of antibodies and F(ab′)_2_ fragment preparations

In a live virus neutralisation assay, all preparations demonstrated activities against both the alpha (Victoria) and omicron (BA.2) strains of SARS-CoV-2 (Table 1). The neutralisation activity increased with the refinement of the preparations, with the F(ab′)_2_ fragments demonstrating the strongest activity. To determine whether the preparations would recognise spike proteins from other SARS-CoV-2 variants, further testing was conducted using the receptor binding domain (RBD) and whole spike protein from alpha (B.1.1.7), beta (B.1.351) and gamma (P.1) strains. Results showed strong ACE2-binding across the strains tested (Table 2). In addition to neutralisation testing, other functional activities were assessed. Antibody-dependent complement deposition assays and neutrophil phagocytosis assays demonstrated activity in the antibody preparations, but when the Fc fragment was removed the complement activity was reduced and there was no evidence of any opsonophagocytic effects (Table 3).

**Table 1:** Neutralisation activity of two divergent strains of SARS-CoV-2 by purified antibodies and F(ab′)_2_ fragments.

SARS-CoV-2 strain	Preparation	Geometric mean IC_50_ (range) (μg/mL) (n = 3)
Victoria (Wuhan-like virus)	Purified antibody	0.57 (0.347–1.198)
	Affinity-purified antibody	0.11 (0.092–0.161)
	F(ab′)_2_ fragment	0.06 (0.027–0.108)
BA.2 (Omicron)	Purified antibody	10.85 (6.934–25.189)
	Affinity-purified antibody	1.95 (0.868–5.138)
	F(ab′)_2_ fragment	0.89 (0.302–2.178)

Data shows IC_50_ values with overall geometric mean IC_50_ and ranges.

**Table 2:** Recognition of antigens from SARS-CoV-2 variants by IgG and F(ab′)_2_ preparations.

Antibody:	Purified antibody	Affinity-purified antibody	F(ab')_2_fragment	Purified antibody	Affinity-purified antibody	F(ab')_2_fragment
Antigen	Spike			RBD		
Wuhan (“WT”)	5848	1259	1093	9295	1194	749.2
Alpha (B.1.1.7)	6935	1318	1235	8669	1699	1425
Beta (B.1.351)	14,004	1822	2372	17,871	2535	4125
Gamma (P.1)	12,002	2029	2048	15,735	2952	3286

Data show IC_50_ values of ACE2-binding for each preparation in ng/mL.

**Table 3:** Complement deposition and phagocytosis functional activity of IgG and F(ab′)_2_ preparations.

	Complement (CAU/mg)	Phagocytosis (OU/mg)
Purified IgG	108.1	219.14
Affinity-purified IgG	218.3	918.18
F(ab′)_2_	67.6	0

*CAU* complement activating units, *OU* opsonophagocytosis units.

### Protective effects of antibodies and F(ab′)_2_ fragments against SARS-CoV-2 disease after a single dose before challenge

**Figure 3:**
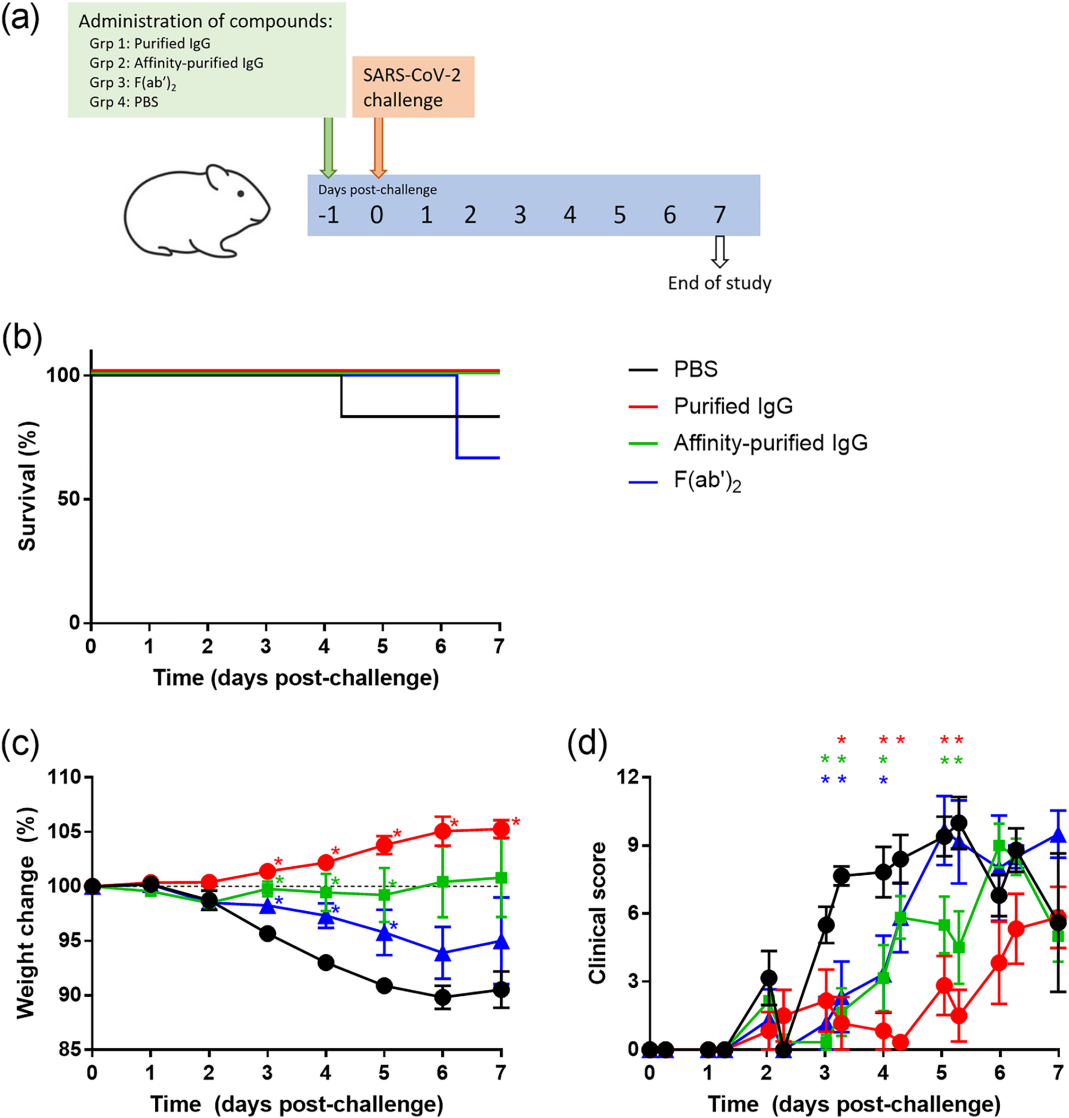
Testing of protective responses of antibody and F(ab′)_2_ preparations against SARS-CoV-2 in hamsters. (**a**) Outline of study schedule, with n = 6 hamsters per group. (**b**) Kaplan–Meier survival plot. (**c**) Body weight changes in animals as a percentage compared to the weight on the day of challenge. (**d**) Clinical score of animals. (**c,d**) Lines show mean values with error bars denoting standard error. *Indicates a statistically significant difference compared to the PBS control group (P < 0.05, Mann–Whitney test).

To ascertain the activity of the antibody-based preparations to protect against SARS-CoV-2 infection, administration to hamsters was undertaken prior to challenge with SARS-CoV-2 (Fig. 3a). Two animals in the F(ab′)_2_ group and one in the PBS group met humane clinical endpoints (Fig. 3b), but these did not reach statistical significance (P > 0.05, Log-Rank survival). In the PBS control group, weight loss was observed whereas those receiving antibody-based preparations had significantly lower loss or no reduction at all on days 3–5 post-challenge (P < 0.05, Mann–Whitney test) (Fig. 3c). In the purified IgG group, this significance extended to the end of the study on day 7 post-challenge. Clinical scores were assessed twice daily, with animals receiving compounds showing significantly lower scores on days 3–4 post-challenge and for day 5 for the purified and affinity-purified antibody groups (P < 0.05, Mann–Whitney test) (Fig. 3d).

### Circulating antibody and F(ab′)_2_ levels at the time of challenge

**Figure 4:**
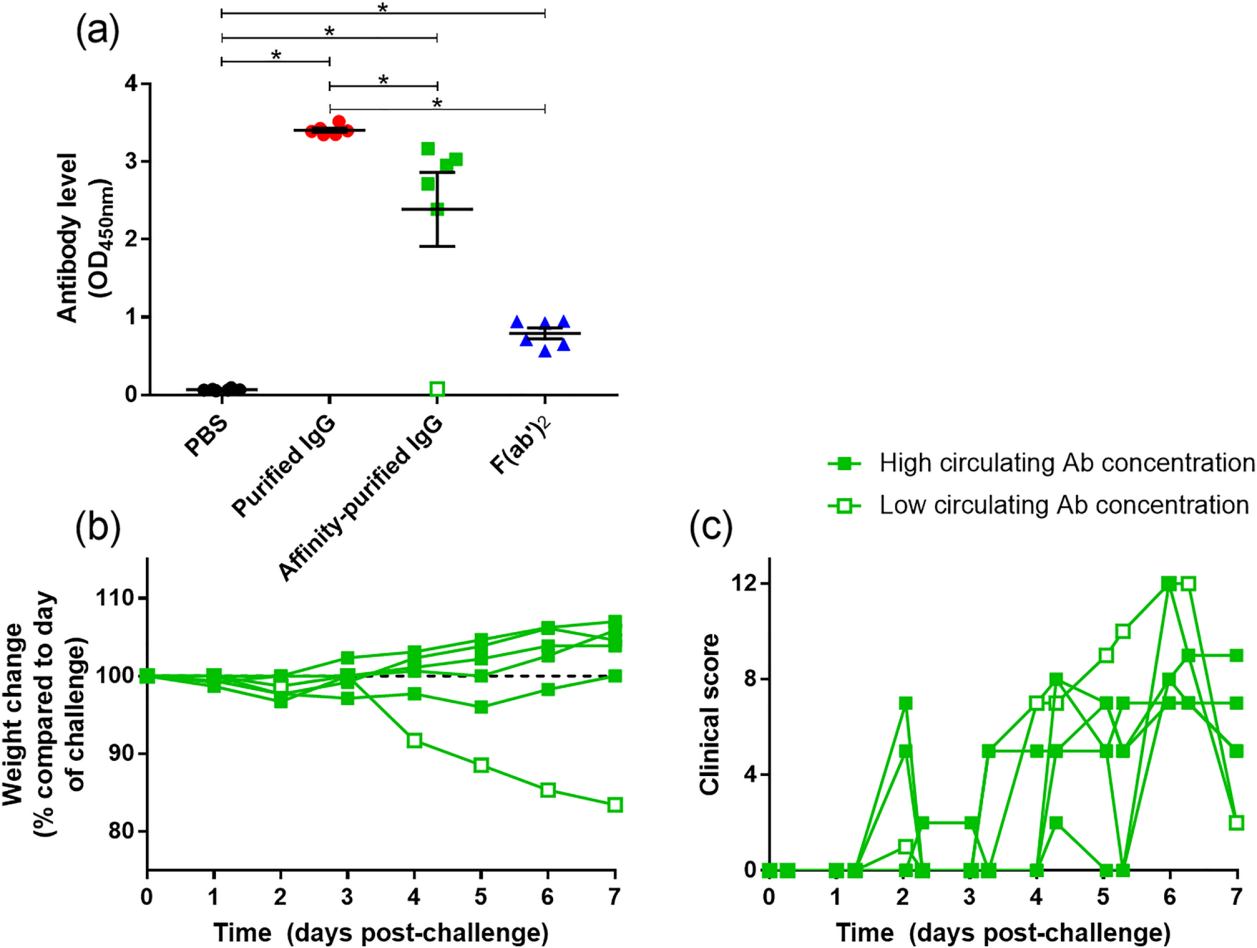
Circulating antibody levels on the day of challenge and comparison of clinical outputs with an animal in which antibody was not detected in circulation. (**a**) Antibody binding to whole spike protein. Results show the mean absorbance level from each animal tested at a 1:100 dilution. Bar and whisker plots denote mean and standard error. *Indicates statistical significance (P < 0.05, Mann–Whitney test). (**b**) Body weight change and (**c**) Clinical score of individual animals receiving affinity-purified antibody, with the individual animal with undetectable levels represented by open symbols.

To determine the levels of compound in the circulatory system, serum samples collected on the day of challenge were evaluated. Results showed high levels in the groups receiving purified and affinity-purified antibodies preparations, but lower levels in the F(ab′)_2_ group (Fig. 4a). One animal in the group receiving affinity-purified antibody had no demonstrable circulating antibody; subsequently, this hamster was compared with the other individual animals from this group. Results revealed increased weight loss in this animal (Fig. 4b), but the clinical score remained similar to other animals in the group (Fig. 4c).

### Pharyngeal viral loads in hamsters receiving antibody or F(ab′)_2_ prior to challenge with SARS-CoV-2

**Figure 5:**
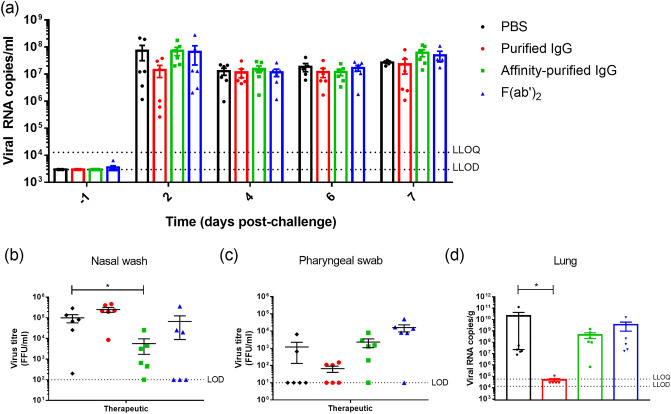
Viral load levels in samples from animals receiving antibody-based compounds prior to challenge with SARS-CoV-2. (**a**) Viral RNA in pharyngeal swabs. Bars show mean values with error bars denoting standard error. (**b**) Viral titre responses from nasal wash samples and (**c**) pharyngeal swab collected 2 days post-challenge. Bar and whisker plots denote mean and standard error. (**d**) Viral RNA levels in lung samples collected at the time of necropsy. Bars show mean values with error bars denoting standard error. * indicates statistical significance (P < 0.05, Mann–Whitney test).

Pharyngeal swab samples were collected every other day post-challenge and tested by PCR to look at viral RNA levels. Significant differences in RNA levels were not observed between those receiving antibody-based compounds compared to the PBS control group (Fig. 5a). On day 2 post-challenge, a nasal wash sample and pharyngeal swab sample were tested for the presence of live virus. In the nasal wash, there was a significant reduction in the group receiving affinity-purified IgG (P < 0.05, Mann–Whitney test) (Fig. 5b), but no significant differences were observed in the pharyngeal swabs (Fig. 5c). At necropsy, viral RNA levels in lung were assessed, with a significant reduction in viral RNA levels observed in the animals which received purified IgG (P < 0.05, Mann–Whitney) (Fig. 5d).

### Histopathological changes associated with prophylactic delivery of antibody-based compounds

**Figure 6:**
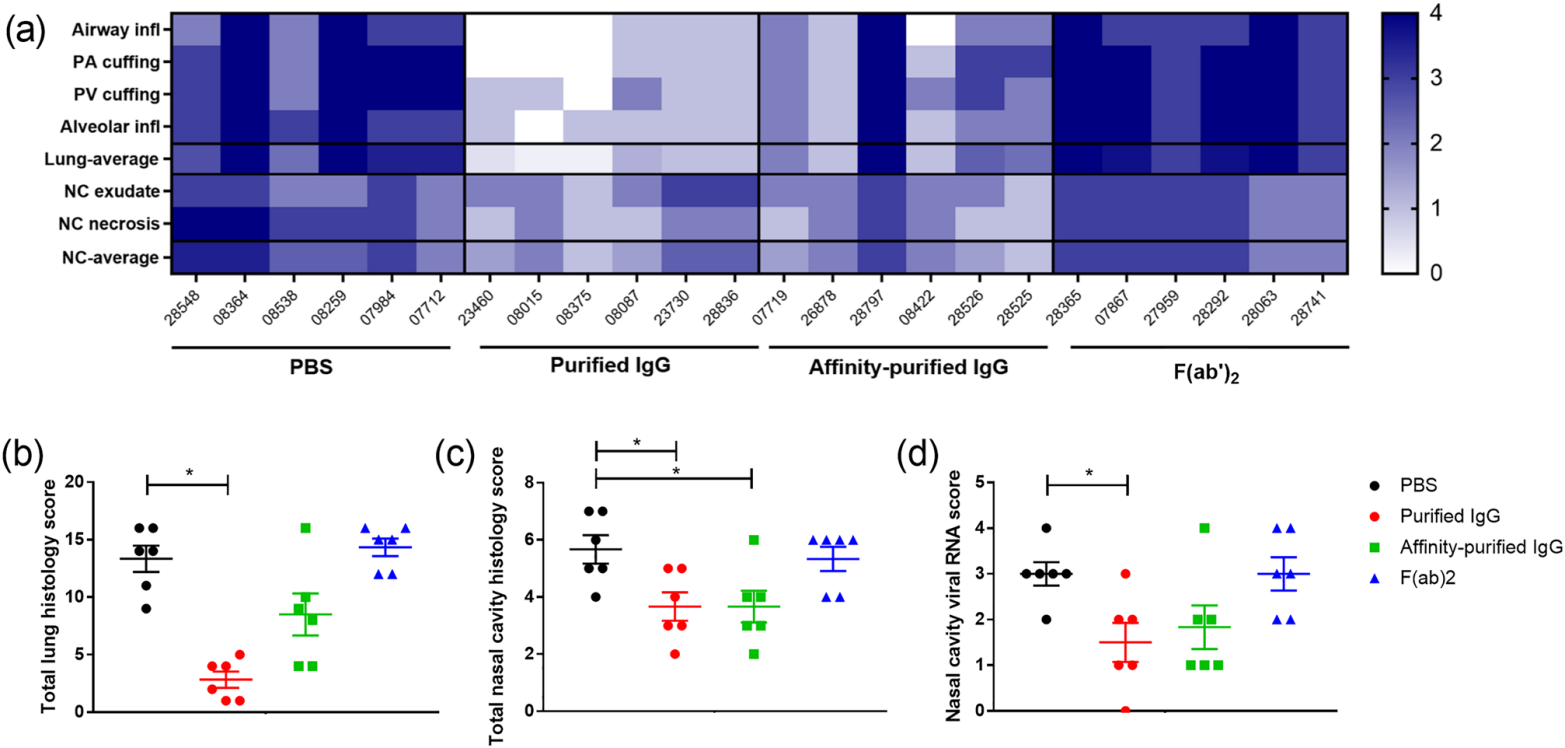
Histopathological changes in the lung and nasal cavity of animals receiving antibody-based compounds prior to SARS-CoV-2 challenge. (**a**) Heatmap showing the severity scores of individual histopathological changes and average scores in the lung and nasal cavity (subjective scoring). *Airway infl* infiltration of airways by inflammatory cells, *PV* peri-vascular inflammatory cell cuffing, *PA* peri-airway inflammatory cell cuffing, *alveolar inflam* infiltration of alveolar spaces and wall by inflammatory cells, *NC exudate* inflammatory cell exudate in nasal cavity lumen, *NC necrosis* epithelial cell degeneration and necrosis in the nasal cavity, (**b**) Total histopathological scores of changes in the lung and (**c**) nasal cavity (subjective scoring). (**d**) The extent of staining of viral RNA in the nasal cavity (quantitative analysis). (**b–d**) Bar and whisker plots denote mean and standard error. *Indicates statistical significance (P < 0.05, Mann–Whitney test).

Lesions consistent with infection with SARS-CoV-2 were observed with varying severity in the lung and nasal cavity of animals from both the test and control groups. In the lung, lesions typically comprised a broncho-interstitial pneumonia with areas of consolidation. Large numbers of inflammatory cells, primarily macrophages and neutrophils with some lymphocytes and plasma cells, infiltrated alveolar spaces, with cell damage and loss; prominent type II alveolar hyperplasia was noted in some areas, alongside alveolar oedema. The airways were also infiltrated by similar inflammatory cells. Whilst larger airways contained fewer inflammatory cells, changes in smaller airways were of increased severity, with concomitant epithelial degeneration and loss. Airways were variably surrounded by lymphocytes and other inflammatory cells. Lymphocytes were also noted surrounding blood vessels and occasionally infiltrating the walls. The severity of microscopic changes varied between groups (Fig. 6a) with a statistically significant reduction in total scores noted for the group which received purified antibody compared to the PBS control group (P < 0.05, Mann–Whitney test) (Fig. 6b). In addition, viral RNA was detected in one animal from each of the PBS group and F(ab′)_2_ groups; lung tissue from the remaining animals in all four groups was negative.

In the nasal cavity, lesions were characterised by variable degeneration and necrosis of the epithelium in the respiratory and olfactory mucosa; and the presence of luminal exudates, comprising mucous and proteinaceous fluid and mixed with inflammatory cells, mainly degenerate neutrophils with some mononuclear cells. There was a statistically significant reduction in total scores for groups which received either the purified antibody or the affinity-purified preparation compared to the PBS group (P < 0.05, Mann–Whitney test) (Fig. 6c). Viral RNA staining was observed in the nasal cavity, with significantly lower levels in the animals receiving purified IgG compared to the PBS control group (P < 0.05, Mann–Whitney test). Significant differences were not observed between the PBS control group and those receiving the affinity-purified or F(ab′)_2_ preparations (Fig. 6d).

**Figure 7:**
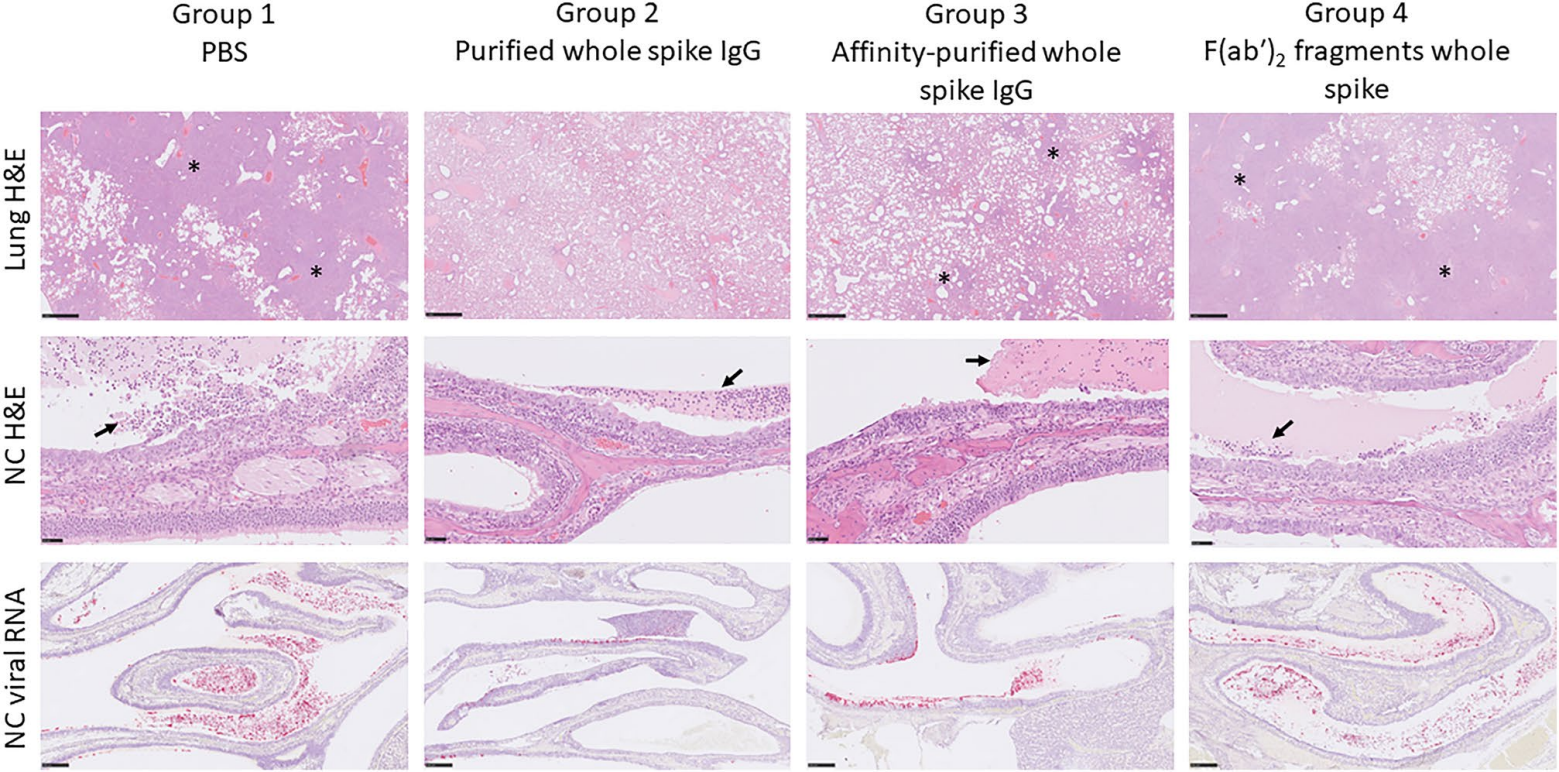
Representative images of microscopic changes in the lung and nasal cavity. Top row, lung- multifocal to patchy areas of pneumonic consolidation (asterisks) (H&E); middle row, nasal cavity – patchy to diffuse inflammation and degeneration of the mucosa with variable luminal exudate (arrows) (H&E); lower row, nasal cavity staining for SARS-CoV-2 viral RNA in the mucosa and luminal exudate (ISH).

Representative images of the severity of microscopic changes in the lung and nasal cavity are shown in Fig. 7.

### Evaluation of the protective effects of F(ab′)_2_ with multiple administrations

**Figure 8:**
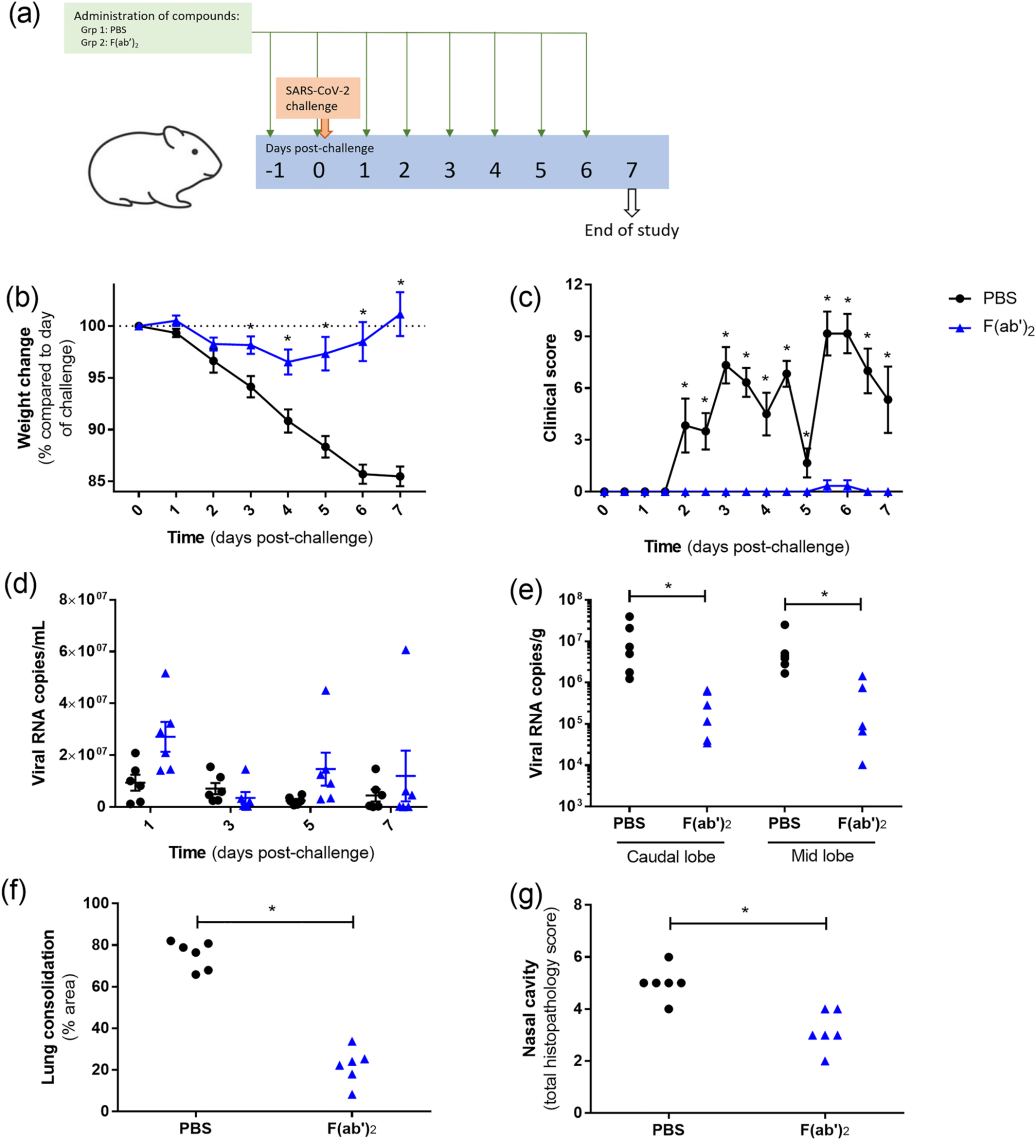
Clinical, virology and histopathological results from animals receiving daily intraperitoneal F(ab′)_2_ preparations. (**a**) Schematic overview of study design. (**b**) Change in body weight and (**c**) clinical score. Lines show mean values with errors bars denoting standard error. (**d**) Viral RNA levels from pharyngeal swabs. Box and whisker plot show mean value and standard error. (**e**) Viral RNA levels from lung samples. (**f**) Percentage of area of lung with consolidation as determined by image analysis. (**g**) Histopathology scores for nasal cavity. *Indicates statistical significance (P < 0.05, Mann–Whitney test).

Due to the detection of lower levels of F(ab′)_2_ in the circulation at the time of challenge compared to whole immunoglobulin preparations, a further study was conducted using a repeated dosing regimen. Hamsters received a dose of F(ab′)_2_ on the day before challenge and then daily throughout the course of the study (Fig. 8a). In this experimental set-up, the clinical outcomes after SARS-CoV-2 challenge were significantly improved in those receiving F(ab′)_2_. After a small (< 5%) loss in body weight, this had recovered by day 7 in those receiving F(ab′)_2_ compared to the PBS control group (Fig. 8b). In addition, apart from signs of ruffled fur in a single animal in the F(ab′)_2_ group at 2 timepoints, no other clinical signs were reported in contrast to sustained clinical scores in those receiving the PBS control (Fig. 8c). Pharyngeal swabs were measured for viral RNA levels, with similar responses between the two groups (Fig. 8d). On day 7 post-challenge, animals were necropsied, and samples collected for viral load analysis and histological examination. Viral RNA levels in the lung were significantly lower in animals which received F(ab′)_2_ across both the caudal and mid areas (Fig. 8e). Histological analysis demonstrated significantly lower lung consolidation and histopathological scores in the nasal cavity in the F(ab′)_2_ group compared to those receiving the PBS control (Figs. 8f,g).

## Discussion

The results presented within give an account of the activities of ovine anti-SARS-CoV-2 spike protein whole IgG compared to a refined F(ab′)_2_ preparation. The results of the live neutralisation assay testing showed that our ovine F(ab′)_2_ had a geometric mean 50% inhibitory concentration (IC_50_) of 0.06 μg/mL. This is comparable with similar equine F(ab′)_2_ candidates which demonstrated an IC_50_ of 0.07 μg/mL^11^. In addition, our ovine F(ab′)_2_ preparation demonstrated ACE2-binding inhibition activity against a range of SARS-CoV-2 strains, including Alpha, Beta, Gamma and Omicron. Whilst equine F(ab′)_2_ fragments have also exhibited neutralising activity across multiple strains, these only went up to Delta variants^26^. Our work with the Omicron BA2 strain, the latest variant at time of writing, extends the knowledge of neutralising efficacy with our ovine-sourced candidate. This cross-reactivity showcases the promising potential of this therapeutical approach.

Whilst neutralisation of virus is likely a major mechanism of action, being multivalent, both IgG and F(ab′)_2_ may also form antibody-target complexes which may be eliminated by phagocytosis^28^. However, we show that there was no evidence of phagocytic activity of the ovine F(ab′)_2_ preparation in contrast to that of whole IgG. Both IgG and F(ab′)_2_ exhibited complement deposition, in agreement with other reports^29^. Whilst this may enable an alternative antiviral mechanism, there is a potential risk that this may also induce anaphylactic reactions via complement activation^4^, thus further study is required.

To determine their effect against disease, the IgG preparations and F(ab′)_2_ were tested in a challenge model of SARS-CoV-2 infection. In an initial study where animals received intraperitoneal antibodies the day before challenge, the whole IgG preparations resulted in a better clinical outcome compared to those receiving F(ab′)_2_ fragments. Differences in the degree of protection have been reported with similar strategies using this approach for antitoxins against ricin, with whole IgG performing better after a single administration; this is likely due to F(ab′)_2_ not maintaining sufficiently high concentrations in the blood to provide protection^30^. The finding that the levels of circulating F(ab′)_2_ were significantly lower than that of whole IgG is due to their variable molecular mass giving different pharmacokinetic profiles^13,31,32^. F(ab′)_2_ produced from equine sources have shown a plasma half-life of approximately 47 h^26^. A second study was undertaken in which F(ab′)_2_ was delivered daily to counteract the lower concentrations in the circulatory system after administration. As conditions were identical to the previous study, and to reduce the number of animals used according to NC3Rs criteria, the IgG preparations were not repeated for this experiment. Results from this study showed significant benefits against clinical disease progression and virus levels, especially in the lung where both viral RNA levels and lung consolidation were significantly lower than in mock-treated animals. These results demonstrate that F(ab′)_2_ may offer positive effects against SARS-CoV-2 infection, although further work will be required to determine a therapeutic effect as dosing started prior to virus challenge in this study.

For the whole IgG preparations, our results demonstrated that purified IgG resulted in reduced severity of pathological changes, and reduced lung viral RNA compared to affinity-purified IgG. This finding was unexpected and indicates that non-specific antibodies may play a role in disease manifestation. As the sheep are kept outside, they will have likely been exposed to a broad range of stimuli resulting in IgG production. Before initiation of the study the sheep were screened for evidence of exposure to Pestiviruses (such as border virus), Blue tongue, Brucella ovis and Q-fever; but may have had exposure to other indigenous pathogens. The sheep were also vaccinated against Johnes disease with a multivalent vaccine (Glanvac 6 in 1 or equivalent), to control five clostridial diseases and Caseous Lymphadenitis, which may have provided extra immuno-stimulatory properties. Protection against SARS-CoV-2 has been suggested with non-specific vaccination approaches, such as with BCG vaccine against tuberculosis^33^. This approach was tested in a non-human primate model and showed that BCG vaccination induced heterologous immune mechanisms which could contribute to moderating SARS-CoV-2 disease severity, although there was no evidence of enhanced viral clearance or reducing disease pathology^34^.

Whilst monoclonal antibodies are undoubtedly a concise therapy, and can be synthetically manufactured, they are inherently susceptible to escape mutations. This has been found with the clinically approved bamlanivimab which lost reactivity against the Delta variant^35,36^. The benefits of polyclonal antibodies recognising a diverse breadth of epitopes across the spike protein thus render them much less likely to evade evolutionary changes. Clinical development of polyclonal antibody-based therapies arising from hyperimmune plasma is feasible and achievable, as evidenced with their use as antivenoms^18^. In addition, the World Health Organization (WHO) has published guidelines for the production, control and regulation of animal-derived immunoglobulin for use as antivenoms^37^ and there is an European Pharmacopoeia monograph on immunosera for human use, animals (0084); therefore an approval strategy exists which is transferrable to other applications, such as infectious disease therapies.

Whilst tested alone during this developmental phase, it is likely that in clinical settings antibody-based therapeutics will be given alongside other treatments. For avian H5N1 influenza, a F(ab′)_2_ product demonstrated synergistic effects when administered alongside the widely used anti-influenza therapy, oseltamivir^38^. Similarly, a rabies F(ab′)_2_ fragment also synergises with existing post-exposure prophylaxis, is well tolerated, and increases efficacy^39^.

In summary, our work demonstrates the refinement of ovine whole IgG into a F(ab′)_2_ preparation; with removal of the Fc region likely reducing several adverse reactions associated with whole antibodies. Further preclinical work is now required to optimise dosing strategies and assess therapeutic usage before consideration for clinical development.

## Methods

### Recombinant proteins

Full-length SARS-CoV-2 spike glycoprotein was produced in Chinese Hamster Ovary (CHO) cells with a His-tag incorporated (REC31868; Native Antigen Company, UK). Similarly, subunit SARS-CoV-2 S1 and S2 proteins were also produced in CHO cells (REC31869 and REC31870, respectively; Native Antigen Company, UK).

### Animals

Border Leicester Cross Merino ewes, aged 12 months or older and born in Australia, were obtained from an approved supplier and kept at a controlled farm registered with the Australian Department of Agriculture. The farm operates under an extremely high health status and standards of animal welfare, with the animals receiving health inspections and veterinary checks weekly and within 3 days of blood sampling.

Golden Syrian hamsters, aged 7–9 weeks (weight range 107–177 g), were obtained from a UK Home Office accredited facility (Envigo RMS UK Ltd). Animals were housed in cages in accordance with the requirements of the UK Home Office Code of Practice for the Housing and Care of Animal Used on Scientific Procedures (1986). During procedures with SARS-CoV-2 animals were housed in a flexible-film isolator within a Containment Level 3 facility. Animals were randomly assigned into groups, with equal allocation of male and female animals. Group sizes of 6 hamsters were used as the minimal number required for statistical significance to be achieved. Animals were sedated before performing nasal washes. Access to food and water was ad libitum and environmental enrichment was provided. All experimental work was conducted under the authority of a UK Home Office approved project licence that had been subject to local ethical review at Public Health England (now part of the UK Health Security Agency [UKHSA]) Porton Down by the Animal Welfare and Ethical Review Body (AWERB) as required by the Home Office Animals (Scientific Procedures) Act 1986. Humane clinical endpoints were determined by 20% body weight loss or severe signs of disease/distress, with animals reaching these limits being euthanised.

### Hyperimmune plasma and purified IgG production

Plasma and IgG production was conducted by International Therapeutic Proteins Ltd. Six sheep were immunised on a 28-day schedule with 0.5 mg recombinant full-length spike protein delivered by subcutaneous injection across six sites: axillae (× 2), inguinal regions (× 2) and supra-scapula (× 2). Freund’s Complete Adjuvant was used for the first immunisation and Freund’s Incomplete Adjuvant subsequently. After a priming period that included two immunisations, plasma was collected via plasmapheresis using an automated MCS machine (Haemonetics Corporation, USA). Each collection typically yielded 600 mL plasma which was stored at − 25 °C until further processing. Plasma was collected on a 28-day schedule, 2 weeks following immunisation. The IgG fraction was purified from the hyperimmune plasma by a series of polyethylene glycol (PEG) fractionations and a zinc precipitation. The product was formulated at a concentration of 50–60 g/L in 20 mM sodium acetate/20 mM NaCl buffer.

### Affinity-purification

Affinity-purification of antibodies were undertaken by the Native Antigen Company. Columns were made using a 1:1 mix of recombinant S1:S2 protein at a ratio of ~ 5 mg recombinant protein per ~ 1 g of de-hydrated, cyanogen bromide-activated Sepharose (Cytiva) according to manufacturer’s instructions, resulting in 5 mL final resin per antigen. Unbound antigen was washed out with 3 × 2 column volumes (CV) of 0.1 M NaOAc, pH4.0, 500 mM NaCl, followed by 3 × 2 CV of 0.1 M Tris–HCl, pH8.0, 500 mM NaCl. This wash cycle was repeated twice. For the purification of antibodies, the resins were transferred to 10 mL polypropylene gravity flow columns (ThermoFisher).

The PEG precipitated IgG-fraction was pH-adjusted by the addition of 1 M HEPES, pH8.0 (1.25 mL per 10 mL of original IgG fraction). After centrifugation (10 min, 4000 × *g*, 20 °C), the clarified supernatant was loaded on the respective antigen column equilibrated in 10 mM HEPES pH8.0 with a contact time of approx. 1.5 h. The columns were washed with 10 CV of equilibration buffer, followed by 10 CV of 10 mM HEPES pH8.0, 300 mM KCl. Antibodies were eluted with 100 mM glycine, pH 2.5. Protein- containing fractions were pooled and adjusted to pH7-8 with 1 M Tris–HCl, pH9.0 (1/5th of the original volume) before dialysis into DPBS.

### F(ab′)_2_ fragmentation

Antibody fragmentation was carried out by MicroPharm Ltd. The bulk affinity purified IgG preparation was titrated to pH 3.1 using 0.5 M Hydrochloric acid and heated. Once the temperature reached 28 °C, 1% w/w pepsin was added and the mixture continuously mixed for 30 min. Digestion was terminated by the addition of 0.5 M Sodium Hydroxide, until the solution reached pH 5.7. The terminated digest was clarified prior to concentration and diafiltration to remove low molecular weight digest fragments. The F(ab′)_2_ was then further enriched using anion exchange chromatography, prior to formulation at 2.7 g/L in a glycine buffered saline, containing maltose and PS80.

### Enzyme-linked immunosorbent assay

Nunc MaxiSorp microtitre plates were coated with 2 μg/mL recombinant protein in bicarbonate buffer overnight at 2–8 °C. Plates were washed 3 times with phosphate buffered saline (PBS) containing 0.05% Tween20 (PBST)and blocked for 1 h at 37 °C with blocking buffer (5% skimmed milk powder in PBS). Plates were incubated for 1 h at 37 °C with prediluted samples (sera collected from sheep blood samples or purified antibody preparations); washed with PBST; and incubated with a donkey anti-ovine IgG horseradish peroxidase (HRP) conjugate (Product 713-035-003; Jackson ImmunoResearch, USA) for 1 h at 37 °C. After further washing, TMB substrate was added and the reaction was stopped by the addition of stop solution before reading the optical density at a wavelength of 450 nm.

### Neutralisation assay

SARS-CoV-2 Australia/VIC01/2020 (GISAID accession, EPI_ISL_406844) (Victoria isolate)^40^ was generously provided by The Doherty Institute, Melbourne, Australia and was subsequently passaged in Vero/hSLAM cells [ECACC 04091501] (European Collection of Cell Cultures, UK) to produce a working stock at passage 4 (P4), at the UKHSA, Porton Down, UK. SARS-CoV-2 lineage BA.2 (Omicron) was isolated at UKHSA, Porton Down from a nasopharyngeal swab taken from a UK patient. Whole genome sequencing was performed on the stock (passage 3) used in this assay.

A microneutralisation assay using Vero-E6 cells (ECACC 85,020,206) and immunostaining protocols, as described previously^41^ was used to assess the neutralising activity of the ovine antibodies, with the following modifications. Antibodies were diluted twofold over a 12-step dilution range (50 µg/mL–0.02 µg/mL), in duplicate (technical replicates). The total incubation time for the Victoria isolate was 24 h whereas for Omicron BA.2 it was 26 h. Immunostaining for both SARS-CoV-2 viruses was performed with anti-nucleocapsid antibodies.

Infectious viral foci were counted with an ImmunoSpot^®^ S6 Ultra-V 367 analyser with BioSpot counting module (Cellular Technologies Europe, Germany). The counted foci data were then imported into R- Bioconductor. Three independent experiments were performed for each antibody. The internal positive control for the Victoria isolate was convalescent plasma, donated to UKHSA from the Northern Ireland Blood Transfusion Service (NIBTS), from a patient recovering from a Wuhan-like virus. The internal positive control for the Omicron BA.2 variant was convalescent plasma, donated to UKHSA, from a patient recovering from Omicron infection.

A midpoint probit analysis compiled using the R Statistical programme (version 3.6.1) was used to determine the amount (μg/mL) of antibody required to reduce SARS-CoV-2 viral foci by 50% (IC_50_) compared with the virus only control (n = 10).

### MSD ACE2-binding inhibition assay

Samples were tested for their ability to inhibit ACE2-binding to different variants of concern (VOC) using the MesoScale Discovery ACE2 assays. A twofold dilution series of the antibodies was assessed on the MSD Plate 7 (K15440U) which harbours full-length spike antigens and RBDs specific to Wuhan and different VOC: Alpha (B.1.1.7), Beta (B.1.351) and Gamma (P.1). Samples were incubated according to the manufacturer’s instructions. Results are reported in inhibiting antibodies (ng/mL).

### Antibody-dependent complement deposition (ADCD) assay

SPHERO carboxyl magnetic blue fluorescent beads (Spherotech, USA) were coupled with SARS-CoV-2 whole spike protein (Lake Pharma, 46,328) using a two-step sulpho-NHS/EDC process^42^. Spike protein was introduced at saturation levels and coupling confirmed by the binding of IgG from a COVID-19 convalescent donor known to have high levels of anti-spike protein IgG. Heat-inactivated NIBSC Anti-SARS-CoV-2 Antibody Diagnostic Calibrant (NIBSC, 20/162) at an initial 1:40 dilution (10 µl sera into 30 µl blocking buffer (BB); PBS, 2% bovine serum albumin (BSA)) followed by a 1:10 dilution into BB) with an assigned arbitrary unitage of 1000U/mL was added in duplicate and serially diluted 2:3 in BB. Affinity-purified ovine polyclonal antibodies (3 µl in duplicate) were added to 27 µl BB and serially diluted 1:3 in BB. This was followed by 20 µl of SARS-CoV-2 spike protein-coated magnetic beads (50 beads per µl) to give a final 1:3 serial dilution range starting at 1:20. The serial dilution for NIBSC 20/162 standard started at 1:80. The mixture was incubated at 25 °C for 30 min with shaking at 900 r.p.m. The beads were washed twice in 200 µl wash buffer (BB + 0.05% Tween-20), then resuspended in 50 µl BB containing 10% IgG- and IgM-depleted human plasma^43^ and incubated at 37 °C for 15 min with shaking at 900 r.p.m. Beads were next washed twice with 200 µl wash buffer and resuspended in 100 µl fluorescein (FITC)-conjugated rabbit anti-human C3c polyclonal antibody (Abcam) diluted 1:500 in BB and incubated in the dark. After two more washes with 200 µl wash buffer, the samples were resuspended in 40 µl HBSS and analysed using an iQue Screener Plus^®^ with iQue Forecyt^®^ software (Sartorius, Germany). For each sample, a minimum of 100 beads were collected. Conjugated beads were gated based on forward scatter and side scatter and then further gated by allophycocyanin (APC) fluorescence. The APC fluorescent-bead population was gated and measured for FITC Median Fluorescent Intensity, which represents deposition of C3b/iC3b. The NIBSC 20/162 calibrant was plotted as a 4 parameter logistic (PL) curve with 1/Y2 weighting and the linear range calculated. The mean fluorescence intensity (MFI) from each sample was interpolated against the NIBSC 20/162 4PL curve and the calculated concentration that hit the linear range was multiplied by the dilution factor to assign activity of the sera as Complement Activating Units (CAU).

### Antibody-dependent neutrophil phagocytosis (ADNP) assay

1 µm crimson-fluorescent carboxylate-modified FluoSpheres™ (Thermo Fisher, F8816) were coupled with SARS-CoV-2 whole spike protein (Lake Pharma, 46,328) using a two-step sulpho-NHS/EDC process^42^. In a 96-well round bottom microtitre plate (Thermo Scientific; 612U96), 20 μL of pre-diluted (ten-point serial dilution from 1:20 to 1:10,240) samples or anti-SARS-CoV-2 antibody diagnostic calibrant reagent (NIBSC; 20/162) were mixed with 20 μL of DPBS-GACM buffer (Dulbecco’s PBS supplemented with 0.1% w/v glucose, 0.5% w/v BSA, 0.9 mM CaCl_2_ and 0.5 mM MgSO_4_ at pH 7.4) containing one million beads. The mix was incubated for 30 min at 37 °C, with shaking at 900 rpm, before the addition of 20 μL of 1:10 diluted IgM- and IgG-depleted human plasma^43^ and 40 μL of DPBS-GACM containing 2.5 × 10^6^/mL granulocyte-differentiated HL-60 cells (ATTC; CCL-240, differentiated with 0.8% N,N-dimethylformamide for 5 days). The plate was incubated for 30 min at 37 °C with shaking at 900 rpm and phagocytosis stopped by placing the plate on ice and adding 80 μL of cold DPBS with 0.02% EDTA. Samples were analysed using an iQue Screener Plus^®^ with iQue Forecyt^®^ software (Sartorius, Germany). Opsonophagocytosis units (OU) were quantified by interpolating the MFI of test samples from a 4PL standard curve of the NIBSC 20/162 calibrant (designated to have 1000 units of anti-SARS-CoV-2 opsonophagocytic activity).

### Protection study designs

In the first study, on the day before challenge 2 mL of purified antibodies (10 mg/mL), affinity-purified antibodies (1 mg/mL) or F(ab′)_2_ fragments (1 mg/mL) were administered to hamsters via the intraperitoneal route.

In a second study, F(ab′)_2_ fragments (1 mg/mL) or PBS was administered in a volume of 1 mL via the intraperitoneal route on the day before challenge and then daily throughout the course of the study.

### Virus challenge

SARS-CoV-2 Victoria/01/2020, described earlier, was used at passage 3. Challenge dilutions were made in sterile PBS with delivery of a total of 5.0 × 10^4^ pfu via intranasal instillation (200 μL total with 100 μL per nare).

### Clinical observations

Animals were monitored twice daily for abnormal clinical signs. These were assigned a score based upon the following criteria: 0, normal; 1, behavioural changes; 2, ruffled fur, dehydrated, wet/stained fur around perineum; 3, arched, wasp-waisted, eyes shut; and 5, dyspnoea (laboured breathing). At the same time each day, animals were weighed.

### Sampling

Pharyngeal swabs were taken on the day of challenge and subsequently on a daily basis. A dry flocked mini-tip swab (product MW002NF; MWE, UK) was used for sampling before adding to 1 mL Virocult universal transport media (product MW951T; MWE, UK). For the first study only, on day 2 post-challenge, a nasal wash was conducted under isoflurane sedation by instillation of 200 μL PBS into each nare with a flexible feeding tube and collection of fluid extract.

At the end of the study, animals were anaesthetised with isoflurane followed by a lethal dose of sodium pentobarbitone delivered via the intraperitoneal route. During necropsy, a sample of lung was collected for viral load analysis. The thoracic pluck (consisting of lung, trachea and associated structures) and the head were immersed in 10% neutral-buffered formalin for histological processing.

### Focus-forming unit (FFU) assay

For the first study, pharyngeal swab and nasal wash samples collected at day 2 were quantified for live virus using a FFU assay. Samples were serially diluted before adding, in duplicate, to a VeroE6 cell monolayer in 96-well flat-bottomed culture plates (seeded 24 h before) for 1 h at 37 °C. Samples were removed and overlay media added, then incubated for 24 h at 37 °C. Plates were fixed overnight by adding 20% formalin and then fumigated before staining using the same techniques as for the neutralisation assay.

### Quantification of viral loads by RT-qPCR

Samples from pharyngeal swabs and lung homogenates were RNA extracted using the BioSprint one-for-all vet kit (Indical, UK) and Kingfisher Flex platform (ThermoFisher, UK). Reverse transcription-quantitative polymerase chain rection of the nucleocapsid (N) gene was used to determine viral loads and was performed using TaqPath™ 1-Step RT-qPCR Master Mix, CG (Applied Biosystems™), 2019-nCoV CDC RUO Kit (Integrated DNA Technologies) and the QuantStudio™ 7 Flex Real-Time PCR platform. Sequences of the N1 primers and probe were: 2019-nCoV_N1-forward, 5’ GACCCCAAAATCAGCGAAAT 3’; 2019-nCoV_N1-reverse, 5’ TCTGGTTACTGCCAGTTGAATCTG 3’; 2019-nCoV_N1-probe, 5’ FAM-ACCCCGCATTACGTTTGGTGGACC-BHQ1 3’, targeting a region of the SARS-CoV-2 nucleocapsid. The cycling conditions were: 25 °C for 2 min, 50 °C for 15 min, 95 °C for 2 min, followed by 45 cycles of 95 °C for 3 s and 55 °C for 30 s. The quantification standard was in vitro transcribed RNA of the SARS-CoV-2 N ORF (accession number NC_045512.2) with quantification between 1 × 10e1 and 1 × 10e6 copies/µl.

### Pathological studies

The samples of thoracic cavity and head remained in 10% NBF for a minimum of seven days. The nasal cavity was decalcified using an EDTA-based solution. The left lung lobe and sagittal section of the nasal cavity were then processed and embedded into paraffin wax. Sections of 4 µm were cut and stained with hematoxylin and eosin (HE) and examined microscopically. In addition, samples were stained using the RNAscope technique to visualise SARS-CoV-2 virus RNA. Briefly, tissues were pre-treated with hydrogen peroxide for 10 min (room temperature), target retrieval for 15 min (98–101 °C) and protease plus for 30 min (40 °C) (Advanced Cell Diagnostics). A V-nCoV2019-S probe (Cat No. 848561, Advanced Cell Diagnostics) was incubated on the tissues for 2 h at 40 °C. Amplification of the signal was carried out following the RNAscope protocol using the RNAscope 2.5 HD Detection kit—Red (Advanced Cell Diagnostics).

All slides were scanned digitally using a Hamamatsu S360 digital slide scanner and examined using ndp.view2 software (version 2.8.24). Sections were examined by a qualified veterinary pathologist who was blinded to the animal and treatment groups. A semi-quantitative histopathology scoring system was used to evaluate microscopic lesions in the lung and nasal cavity (reported elsewhere^27^); in addition, ‘Nikon NIS-Ar’ software (version 5.21.02) was used to perform digital image analysis to calculate the percentage area of pneumonia and quantify the presence of viral RNA in lung sections. For nasal cavity, a semiquantitative scoring system was applied to evaluate the presence of virus RNA: 0 = no positive staining; 1 = minimal; 2 = mild; 3 = moderate and 4 = abundant staining.

### Statistical analysis

Statistical analyses were performed using MiniTab, version 16.2.2 (Minitab Inc). A non-parametric Mann–Whitney statistical test was applied to ascertain significance between groups. A significance level below P = 0.05 was considered statistically significant.

## Data Availability

The datasets generated during and/or analysed during the current study are available from the corresponding author on reasonable request.
