# Publisher Correction: Refinement of an ovine-based immunoglobulin therapy against SARS-CoV-2, with comparison of whole IgG versus F(ab′)_2_ fragments

**DOI:** 10.1038/s41598-023-42526-y

**Published:** 2023-09-18

**Authors:** Stephen Findlay‑Wilson, Linda Easterbrook, Sandra Smith, Neville Pope, Matthew Aldridge, Gareth Humphries, Holger Schuhmann, Didier Ngabo, Emma Rayner, Ashley Otter, Thomas Coleman, Bethany Hicks, Rachel Halkerston, Kostis Apostolakis, Stephen Taylor, Susan Fotheringham, Amanda Horton, Irene CanoCejas, Matthew Wand, Julia A. Tree, Mark Sutton, Victoria Graham, Roger Hewson, Stuart Dowall

**Affiliations:** 1 UK Health Security Agency (UKHSA), Porton Down, Salisbury, Wiltshire SP4 0JG UK; 2 International Therapeutic, Proteins Ltd, Longford, TAS 7301 Australia; 3 International Therapeutic Proteins Ltd, Goleigh Farm, Hampshire, Selborne GU34 3SE UK; 4 MicroPharm Ltd, Station Road, Newcastle Emlyn, SA38 9BY UK; 5 Native Antigen Company, Langford Locks, Kidlington, Oxford, OX5 1LH UK

Correction to: *Scientific Reports* https://doi.org/10.1038/s41598-023-40277-4, published online 25 August 2023

The original version of this Article contained errors in Table 2, where the data in the columns ‘Purified antibody’ and ‘Affinity-purified antibody’ was interchanged for the Antigen ‘RBD’. The correct and incorrect data appears below.

Incorrect:

**Table:** 

Purified antibody	Affinity-purified antibody
RBD	
1194	9295
1699	8669
2535	17,871
2952	15,735

Correct:

**Table:** 

Purified antibody	Affinity-purified antibody
RBD	
9295	1194
8669	1699
17,871	2535
15,735	2952

The original Article has been corrected.

